# Vitamin D Antagonises the Suppressive Effect of Inflammatory Cytokines on CTLA-4 Expression and Regulatory Function

**DOI:** 10.1371/journal.pone.0131539

**Published:** 2015-07-02

**Authors:** Louisa E. Jeffery, Omar S. Qureshi, David Gardner, Tie Z. Hou, Zoe Briggs, Blagoje Soskic, Jennifer Baker, Karim Raza, David M. Sansom

**Affiliations:** 1 Medical Research Council Centre for Immune Regulation, School of Immunity and Infection, Institute of Biomedical Research, University of Birmingham Medical School, Birmingham, United Kingdom; 2 UCL Institute of Immunity and Transplantation, Royal Free Campus, University College London, London, United Kingdom; 3 Department of Rheumatology, Sandwell and West Birmingham Hospitals NHS Trust, Birmingham, United Kingdom; University of Alabama at Birmingham, UNITED STATES

## Abstract

The immune suppressive protein CTLA-4 is constitutively expressed by Tregs and induced in effector T cells upon activation. Its crucial role in adaptive immunity is apparent from the fatal autoimmune pathology seen in CTLA-4 knockout mice. However, little is known regarding factors that regulate CTLA-4 expression and their effect upon its function to remove CD80 and CD86 from antigen presenting cells by transendocytosis. Th17 cells are emerging as significant players in autoimmunity as well as other diseases. Therefore, in this study we have examined the effects of Th17 polarising conditions on CTLA-4 expression and function in human T cells and show that Th17 conditions can suppress the expression of CTLA-4 and its transendocytic function. In contrast to Th17 cells, vitamin D is inversely associated with autoimmune disease. We have previously shown a striking ability of 1,25 dihydroxyvitamin D_3_ (1,25(OH)_2_D_3_) to enhance CTLA-4, however, its effects upon B7 transendocytosis and its activity in the context of inflammation remained unknown. Here we show that induction of CTLA-4 by 1,25(OH)_2_D_3_ can actually be enhanced in the presence of Th17 polarising cytokines. Furthermore, its transendocytic function was maintained such that T cells generated in the presence of Th17 conditions and 1,25(OH)_2_D_3_ were highly effective at capturing CTLA-4 ligands from antigen presenting cells and suppressing T cell division. Taken together, these data reveal an inhibitory effect of Th17 polarising conditions upon CTLA-4-mediated regulation and show that 1,25(OH)_2_D_3_ counteracts this effect. Given the importance of CTLA-4-mediated suppression in the control of autoimmune diseases, our novel data highlight the importance of vitamin D in inflammatory settings.

## Introduction

CTLA-4 is a critical suppressive protein that is expressed constitutively by regulatory T cells (Treg) and is induced on conventional T cells following activation [1–4]. It functions to restrain inappropriate activation of autoreactive T cells and to restore T cell homeostasis following activation. This crucial regulatory role of CTLA-4 is evident from the lethal lymphoproliferative phenotype of CTLA-4 knockout mice [5, 6]. Recently, we and others observed CTLA-4 genetic variants that affected the level of CTLA-4 protein in autoimmunity and immunodeficiency, which indicates the importance of CTLA-4 expression in controlling human disease [7, 8]. Understanding how CTLA-4 expression is regulated therefore holds potential for therapeutic advances in conditions characterised by inappropriate or excessive T cell activation. Numerous studies have investigated the mechanisms by which CTLA-4 functions, leading to a range of proposed models [9–11]. An important feature of CTLA-4 biology is its internalisation and cycling back to the plasma membrane as well as its trafficking to lysosomes for degradation [12–14]. Consistent with this biology, we recently observed that CTLA-4 is able to remove its ligands, CD86 and CD80, from APCs and target them for degradation in a process termed transendocytsosis [15]. In this way, CTLA-4 reduces the availability of its shared ligands for CD28 co-stimulation in a quantitative manner that depends on the level of CTLA-4 expression. Surprisingly, despite the crucial role of CTLA-4 in immune regulation, relatively little is known about how its expression level and transendocytic activity are controlled, including the influence of environmental factors or the cytokine milieu.

Th17 cells, which play an important role in the clearance of certain extracellular and intracellular pathogens [16, 17], are differentiated under inflammatory cytokine conditions and their dysregulation contributes to the pathology of a range of autoimmune diseases [18–20]. Their differentiation is intriguing since it is closely related to that of Treg through the common involvement of TGFβ [21, 22] with cytokines such as IL-1β, IL-6 and IL-23 promoting a Th17 outcome in humans [23, 24]. The relationship between inflammatory Th17 conditions and CTLA-4 expression is currently not well understood. In contrast, vitamin D is emerging as an important regulator of inflammatory responses. Indeed, low vitamin D status is associated with an increased risk of inflammatory diseases, including multiple sclerosis, type 1 diabetes, rheumatoid arthritis and systemic lupus erythematosis (reviewed in [25–29]). Furthermore, vitamin D supplementation in mouse models of autoimmunity has suggested both prophylactic and therapeutic benefit [30–33].

We have shown that production of Th17-related cytokines is inhibited by vitamin D whilst regulatory markers, including CTLA-4, Foxp3 and IL-10, are increased [34] suggesting that vitamin D antagonises inflammatory outcomes and promotes regulation. In order to achieve a regulatory effect in an autoimmune setting, vitamin D would need to be effective within an inflammatory milieu. We therefore sought to determine the impact of such an environment on the expression and function of CTLA-4. We show that pro-Th17 cytokines substantially reduce CTLA-4 expression and function. However, even under Th17 polarizing conditions, vitamin D continues to drive upregulation of CTLA-4, generating T cells with CTLA-4-dependent regulatory function.

## Materials and Methods

This study was approved by the University of Birmingham Ethics Committee and given approval number ERN_14–0446.

### Cell isolation and culture

PBMCs were isolated by Ficoll gradient centrifugation from fresh leukocyte reduction system cones provided by the National Blood Service, Birmingham, UK. PBMCs were washed twice with PBS and twice with MACS buffer (0.5% BSA, 2 mM EDTA in PBS) and re-suspended at 1 x 10^8^ cells/ml for magnetic separation. Conventional CD4+CD25- T cells and CD14+ monocytes were enriched by negative selection using cell separation reagents (StemCell Technologies). Greater than 95% purity was obtained as assessed by flow cytometry.

T cells were cultured in serum free medium (CellGenix) supplemented with 50U/ml Penicillin and Streptomycin (Gibco, Life Technologies). To assess the effect of cytokines and 1,25(OH)_2_D_3_ upon their phenotype, T cells were stimulated with antiCD3CD28 Dynabeads (LifeTechnologies) at a ratio of 1 bead: 4 T cells in the presence of recombinant cytokines and supplements as described in the figure legends. Supplements were added at the following concentrations: IL-1β (10ng/ml, Peprotech), IL-6 (20ng/ml, Immunotools), IL-23 (10ng/ml R and D Systems), TGFβ (1ng/ml, R and D Systems), 1,25(OH)_2_D_3_ (10nM, Sigma Alrich) and anti-human CTLA-4 (ticilimumab) (20μg/ml, a generous gift from Pfizer). The carrier for 1,25(OH)_2_D_3_ was ethanol. It was diluted 1 in 1000 (v/v) into the culture. This concentration did not affect the measured outcomes. For all other reagents the vehicle was PBS.

Monocyte derived dendritic cells (DCs) were cultured from monocytes in RPMI containing 10% FBS (Biosera), 50U/ml Penicillin and Streptomycin, 200μM glutamine (Life Technologies) (RPMI-FBS) and supplemented with GM-CSF (800U/ml, Peprotech) and IL-4 (500U/ml Peprotech). 1x10^6^ cells were plated per well of a 24 well culture plate. At two to three days, cells were supplemented with fresh medium containing IL-4 and GM-CSF and cultured for a further six or seven days before use. DCs were CD11C+, CD14- and up-regulated CD86, CD80, CD40 and HLA-DR upon maturation with LPS (100ng/ml (Sigma Aldrich). All cells were cultured at 37°C, 95% humidity and 5% CO_2_.

### Cell labelling

For some experiments, T cells were labelled with cell trace proliferation dyes, CFDA-SE or cell trace violet (Molecular probes, Life Technologies). Cells were washed two times with PBS and re-suspended in cell proliferation dye. CFDA-SE labelling was performed for 10 minutes at room temperature after which cells were washed three times in RPMI-FBS. For cell trace violet labelling T cells were labelled with cell trace violet for 20 minutes at 37°C. 10% FBS RPMI was added to quench the labelling. After incubation for 5 minutes at 37°C and centrifugation at room temperature, cells were washed a further two times with 10% FBS RPMI.

### Flow cytometry

Dead cells were labelled with near-IR LIVE/DEAD fixable dead cell stain (Molecular Probes, Life Technologies) before fixation. For analysis of total CTLA-4, Foxp3 and CD25, cells were fixed, permeabilised and stained with ebioscience Foxp3 staining buffers according to the manufacturer’s instructions. For analysis of cytokine expression, cells were re-stimulated with PMA (50ng/ml), and ionomycin (1μM) for 5 hours, with Brefeldin A (10μg/ml) present during the last 4 hours (all from Sigma Aldrich). Cells were fixed with 3% paraformaldehyde in PBS for 12 minutes followed by a 5 minute wash with PBS under centrifugation. Cells were then permeabilised with 0.1% saponin (Acros Organics) prepared in PBS and stained with cytokine detection antibodies. Cells were acquired on a Dako Cyan flow cytometer (Dako Cytomation) and data analysed using FlowJo software (Tree Star). All antibodies were purchased from ebioscience or BD Biosciences and expression quantified relative to the appropriate isotype control.

### Real-time PCR

Total RNA was extracted using the TRIzol method (Life Technologies/Invitrogen). A total of 0.5μg was reverse transcribed with random hexamers using TaqMan reverse transcription reagents (Life Technologies/Applied Biosystems). Quantitative real-time PCR for VDR and 18SrRNA was then performed on an Applied Biosystems 7900 machine using assays on demand from Applied Biosystems (18S rRNA, 4319413E; VDR Hs00172113_m1). Amplification of cDNAs involved incubation at 50°C for 2 minutes and 95°C for 10 minutes followed by 40 cycles of 95°C for 15 seconds and 60°C for 1 minute. VDR mRNA expression was then calculated relative to 18SrRNA using the delta Ct method.

### CD86 acquisition assay (transendocytosis)

A Chinese Hamster Ovarian (CHO) cell line expressing green fluorescent protein-tagged human CD86 (CD86-GFP) was generated as previously described [15] and cultured in DMEM (Life Technologies, supplemented with 10% FBS (Biosera), 50U/ml Penicillin and Streptomycin, 200μM glutamine (Life Technologies). Cells were passaged every 3 days. To enable CD86-GFP to be measured exclusively in T cells at the end of the assay, CHO-cells were labelled with 5mM Cell Trace Far Red DDAO-SE tracking dye (Molecular probes, Life Technologies) immediately before use. T cells were prepared by stimulating them for four days with antiCD3/CD28 Dynabeads beads under Th0 or Th17 conditions in the presence or absence of 1,25(OH)_2_D_3_. Beads were removed using a magnet (Easysep StemCell Technologies) and cells washed with PBS and resuspended in serum free medium (CellGenix). A proportion of T cells were cultured with anti-CTLA-4 (40μg/ml) for 30 minutes before assembling the T cell-CHO-CD86GFP co cultures. 50,000 T cells and 150,000 CHO cells were cultured in a 96 well round bottom plate for 4 hours and 0.5μg/ml anti-CD3 (clone OKT3) added to promote CTLA-4 cycling. Control wells to set the background CD86-GFP transfer were prepared in which 5μg/ml anti-CD86 (clone BU63) was included. 30 minutes before analysis, anti-CTLA-4-PE (BD Biosciences) was added to label cycling CTLA-4. Cells were analysed live by FACS. T cells were selected by gating according to forward-scatter/side scatter and single cells selected by pulse width. CHO-cells were excluded by selecting only far-red negative cells. Total CD86 acquisition was calculated by frequency of CD86-GFP+ cells x median fluorescence intensity of CD86-GFP+ cells. Non CTLA-4-mediated CD86-GFP acquisition, as determined from aCTLA-4 blocking antibody cultures, was then subtracted.

### Dendritic Cell CD80/CD86 downregulation assays

Bead stimulated T cells, polarized for six days under Th0 or Th17 conditions in the presence or absence of 1,25(OH)_2_D_3_, were cell trace violet labelled and a proportion treated with anti-CTLA-4 (40μg/ml) for 30 minutes before combining with DCs. T cells and DCs were co-cultured overnight in 96 well flat bottom plates at a ratio of 1DC:10 T cells in the presence of 0.5μg/ml anti-CD3 (OKT3). At 24 hours, cells were stained for CD86, CD80, CD11C and CD40 (BD Biosciences) and analysed by flow cytometry. DCs were selected by scatter and as violet trace negative.

### Suppression assays

T cell suppressors were prepared by stimulating CD4+CD25- T cells with beads for six days. Beads were removed using a magnet (Easysep StemCell Technologies) and suppressors labelled with CFDA-SE (Molecular Probes, Life Technologies) as described above. A fraction of the suppressors were incubated with antiCTLA-4 (40μg/ml) for 30 minutes before the addition of autologous DCs and allogenic, unstimulated violet cell trace labelled CD4+CD25- ‘responder’ T cells. Cells were combined at a ratio of 1DC: 40 responders: 8 suppressors and 0.5μg/ml antiCD3 (OKT3) added. To control for cell number, CFDA-SE labelled unstimulated CD4+CD25- T cells were used in place of suppressors in control cultures. After 5 days, responder cell proliferation was monitored by flow cytometry. Single cells were selected according to pulse width and responder T cells identified as violet trace +ve, CFDA-SE-ve.

### Statistical analysis

GraphPad Prism 5.0a software (GraphPad) was used for graphical summary and statistical analysis was performed using SPSS statistics version 22. Non-parametric Wilcoxon tests were used to test significance between two conditions when multiple treatments had not been used and n>5. To test interactions between 1,25(OH)_2_D_3_ and cytokine treatments repeated measures two factor within subject analysis with Huynf-Feldt correction was performed. For markers that did not show interaction the two factor analysis was re-run in the absence of interaction. Where interaction was detected single factor repeated measures analysis was performed to determine the effect of cytokine treatment under control and 1,25(OH)_2_D_3_ conditions separately. The Shapiro-Wilk normality test and inspection of normal Q-Q plots were used to confirm that the data could be tested with these parametric models. For data sets that did not pass the normality test (IL-17, IFNγ and IL-10) the data were log_10_ transformed, since by this transformation the residuals from the mean became normally distributed.

## Results

### Th17 polarising cytokines reduce CTLA-4 expression

**Fig 1:**
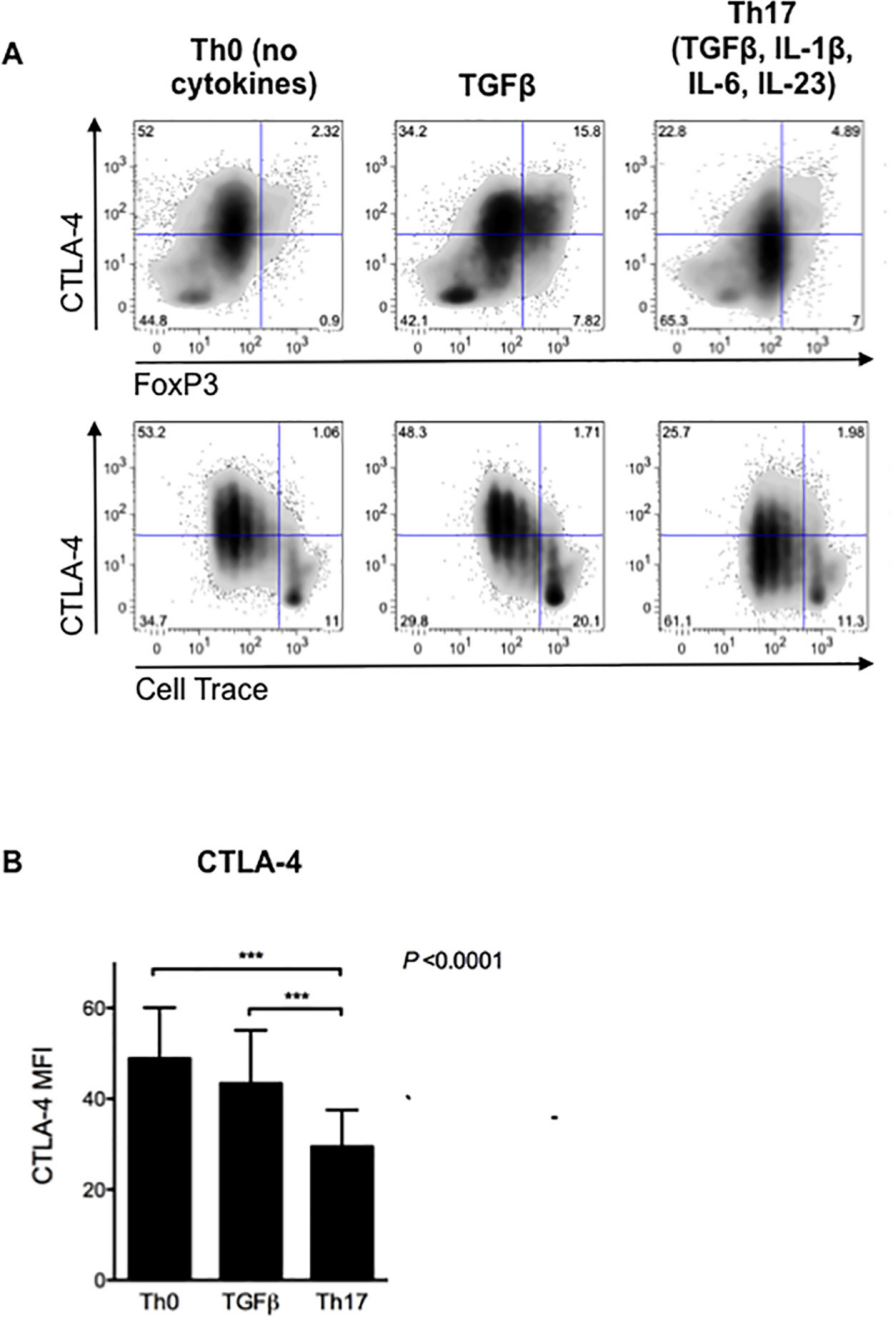
Th17 polarising cytokines reduce CTLA-4 expression. Cell trace-labelled CD4+CD25- T cells were stimulated for four days with antiCD3CD28 beads under no cytokine supplement (Th0), with TGFβ alone or with the pro-Th17 cocktail (TGFβ with IL-1β, IL-6 and IL-23) as indicated and expression of total CTLA-4 and Foxp3 assessed by flow cytometry. **A)** Representative FACS plots showing CTLA-4 against Foxp3 expression and cell division, indicated by cell-trace dilution. **B)** Summary of CTLA-4 expression for 12 donor donors. Bars indicate mean values and error bars show standard deviation. Significance was tested by repeated measures, single factor within subject analysis (* = P<0.05, *** = P<0.001).

To assess the effect of Th17 polarisation on the expression of CTLA-4, we stimulated human CD4+CD25- T cells with anti-CD3/CD28 beads under Th0 conditions (no added cytokines) with TGFβ or with the Th17 polarising cytokines, TGFβ, IL-1β, IL-6 and IL-23, as shown (Fig 1a and 1b). Serum free medium was used to avoid background effects of TGFβ. Despite the absence of serum, the frequency of live cells at the end of culture was 75.4±8.3% and was not influenced by the cytokine treatment (ANOVA, P = 0.52). Whilst addition of TGFβ alone strongly induced Foxp3 it did not affect expression of CTLA-4. However, when TGFβ was combined with the pro-Th17 cytokines, IL-1β, IL-6 and IL-23, which did not affect CTLA-4 without TGFβ (S1 Fig), a significant decrease in CTLA-4 expression was observed (Fig 1). This was not the result of altered kinetics, since CTLA-4 expression was reduced across all divisions as defined by cell trace peaks (Fig 1a).

**Fig 2:**
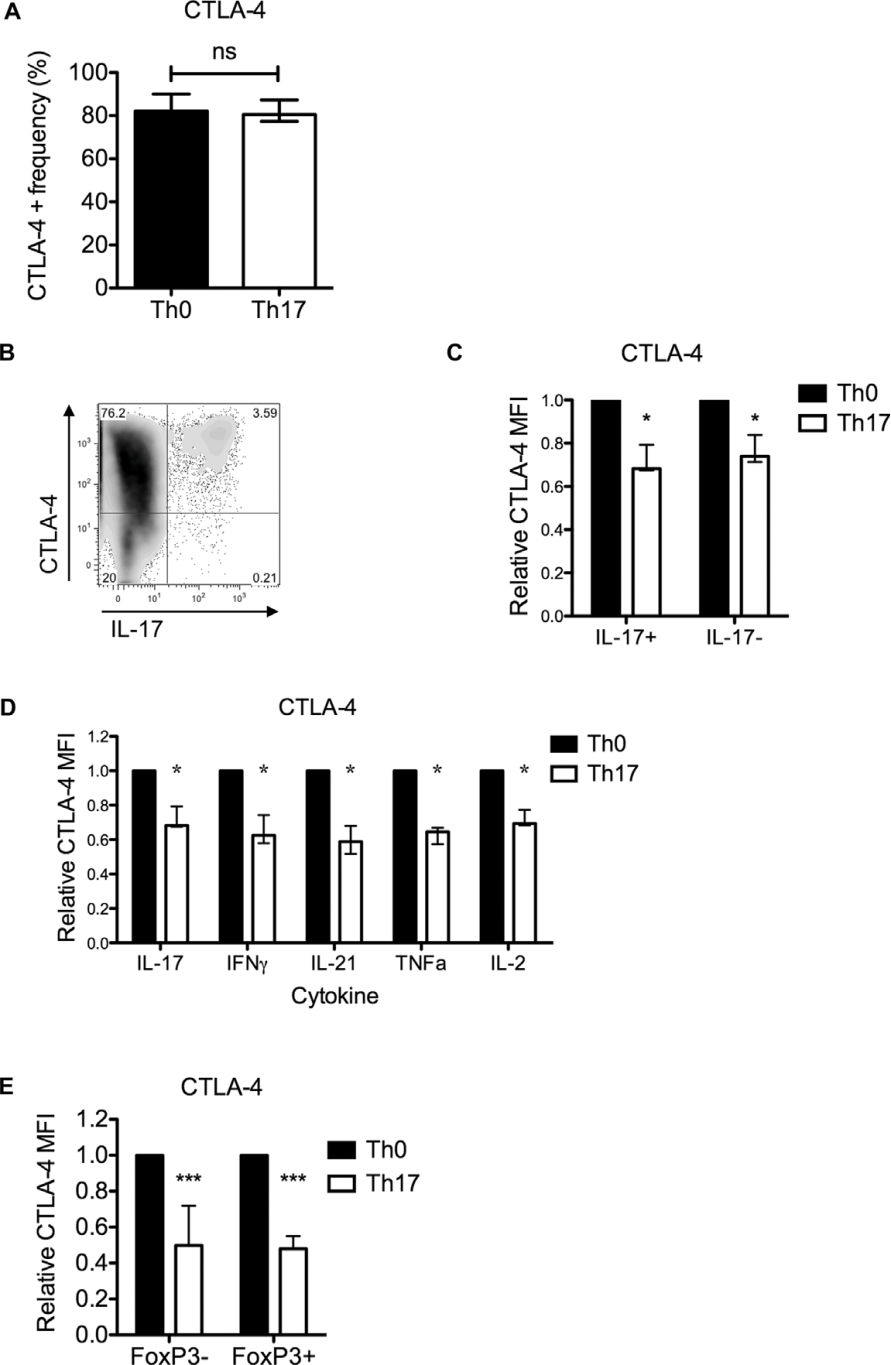
Suppression of CTLA-4 by Th17 polarising cytokines is not specific to IL-17+ T cells. CD4+CD25- T cells were stimulated in the presence of Th17 polarising cytokines for four days and assessed for IL-17, IFNγ, IL-21, TNFα, IL-2 or Foxp3 in combination with CTLA-4 by flow cytometry. **A)** Frequency of total CTLA-4+ cells. **B)** Representative bivariate FACS plot of CTLA-4 versus IL-17 for cells cultured under Th17 polarising conditions. **C)** CTLA-4 expression in CTLA-4+ T cells gated according to IL-17 expression. **D)** CTLA-4 expression by CTLA-4+ T cells that expressed IL-17, IFNγ, IL-21, TNFα or IL-2. **E)** CTLA-4 expression in CTLA-4+ T cells defined by FoxP3 expression. In C, D and E expression under Th17 conditions is expressed relative to the level under Th0 conditions. Data are summarised for n≥7 donors. Bars indicate median values and error bars show the semi interquartile range. Significance with respect to cells expressing the marker under Th0 conditions was tested by Wilcoxon matched paired tests. (* = P<0.05, ** = P<0.01, *** = P<0.001).

To examine whether loss of CTLA-4 expression was a consequence of the Th17 transcriptional program or a more general phenomenon seen in all T cells exposed to Th17 polarising cytokines regardless of differentiation status, we measured CTLA-4 expression in relation to cytokine expression by flow cytometry. CTLA-4 was expressed by approximately 80% of T cells stimulated under either Th0 or Th17 conditions (Fig 2a) and by the majority of cells that expressed IL-17 (Fig 2b). Notably Th17 polarising conditions reduced CTLA-4 expression in CTLA-4+IL-17- as well as CTLA+IL-17+ T cells (Fig 2c), suggesting that the suppressive effect of Th17 polarising conditions upon CTLA-4 is not limited to cells undergoing the Th17 program of differentiation but is a general phenomenon. We explored this further by examining effects of Th17 polarising cytokines on CTLA-4 expression in T cells defined by the expression of other pro-inflammatory cytokines or regulatory-associated FoxP3. Th17 polarising cytokines reduced CTLA-4 in cells expressing IFNγ, IL-21, TNFα and IL-2 (Fig 2d) as well as in FoxP3+ and FoxP3- T cells (Fig 2e). Thus pro-Th17 cytokines suppress CTLA-4 in T cells of different lineages, both regulatory and inflammatory.

### 1,25(OH)_2_D_3_ promotes a Treg phenotype and increases CTLA-4 expression even under inflammatory conditions

**Fig 3:**
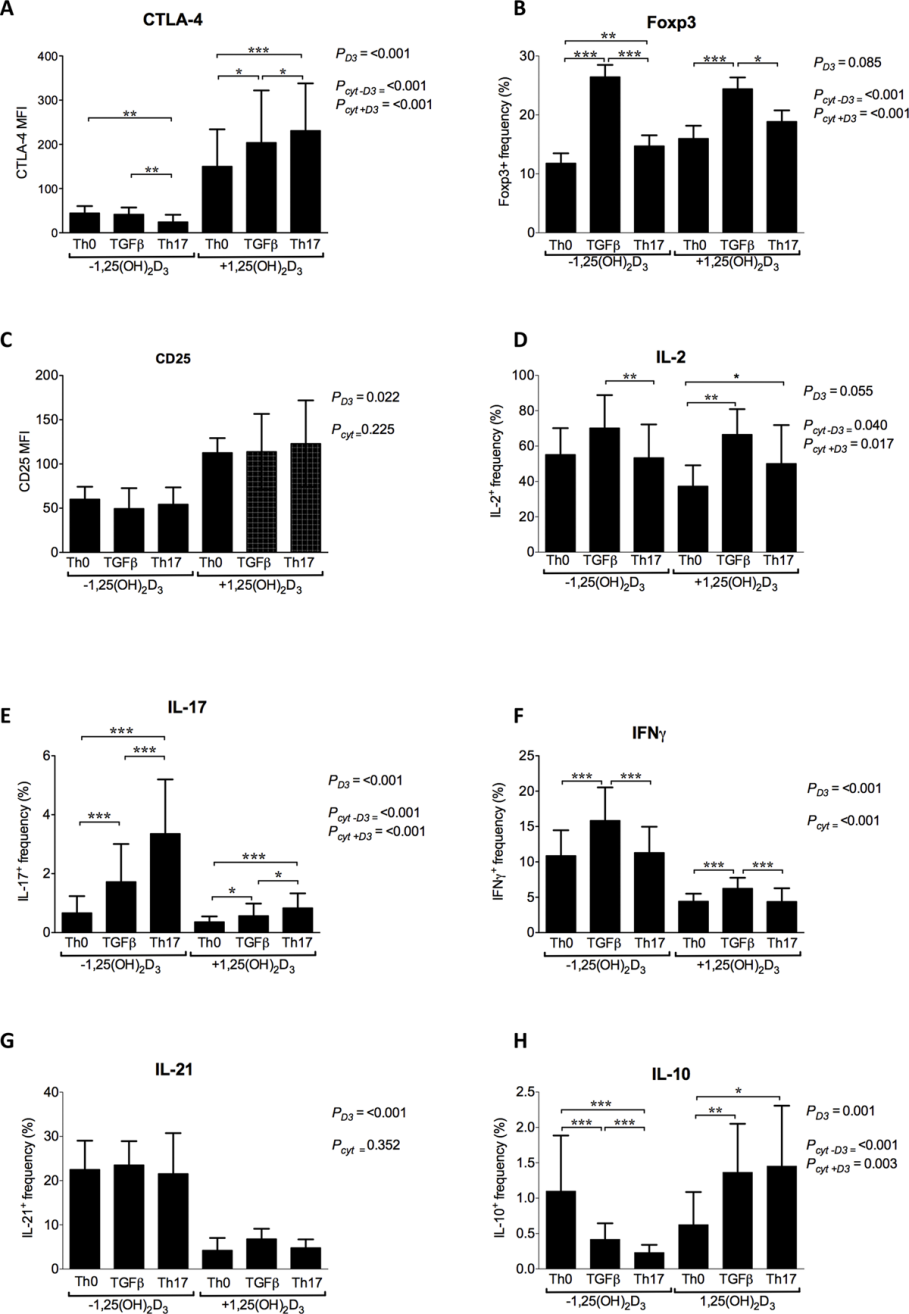
1,25(OH)_2_D_3_ maintains a regulatory T cell phenotype even under inflammatory, Th17 polarising conditions. CD4+CD25- T cells were stimulated in the presence of recombinant cytokines IL-1β, IL-6, IL-23 and TGFβ as indicated with or without 1,25(OH)_2_D_3_ and expression of regulatory-associated markers CTLA-4, Foxp3 and CD25 assessed at four days and cytokines IL-2, IL-17, IFNγ, IL-21 and IL-10 measured at five days by flow cytometry. Data are summarised for n≥5 donors. Bars indicate mean values and error bars show the standard deviation. Repeated measures, two factor within subject analysis was used to test interaction between 1,25(OH)_2_D_3_ and cytokine treatment (S1 Table). For markers that did not show interaction the two factor analysis was re-run in the absence of interaction and P values for each factor are shown (1,25(OH)_2_D_3_ = *P*_*D3*_ and cytokine treatment = *P*_*Cyt*_. Where interaction was detected, single factor analysis was performed. P values are shown for the effect of cytokine treatment under control (*P*_*cyt—D3*_) and 1,25(OH)_2_D_3_ (*P*_*cyt + D3*_) conditions separately. Significant contrasts between cytokine treatments are indicated by stars (* = P<0.05, ** = P<0.01, *** = P<0.001).

Given that we have previously demonstrated that 1,25-dihydroxyvitamin D_3_ (1,25(OH)_2_D_3_) can increase CTLA-4 expression, we next sought to determine if the inhibitory effect of Th17 polarising cytokines on CTLA-4 expression was maintained in the presence of 1,25(OH)_2_D_3_. CD4+CD25- T cells were therefore stimulated as before under Th0, TGFβ alone and Th17 conditions, either in the presence or absence of 10nM 1,25(OH)_2_D_3_. Notably, 1,25(OH)_2_D_3_ did not influence the frequency of live cells at the end of culture under any cytokine treatment (P_D3_>0.05 and P_Cyt_>0.05). However, across cytokine treatments, 1,25(OH)_2_D_3_ promoted marked up-regulation of CTLA-4 expression (Fig 3a and S1 Table). Furthermore a strong interaction between cytokine treatment and 1,25(OH)_2_D_3_ was observed indicating that the cytokine effect is different when 1,25(OH)_2_D_3_ is present. Subsequent inspection using single factor within subject analysis showed that whilst Th17 conditions suppressed CTLA-4 in the absence of 1,25(OH)_2_D_3_, when 1,25(OH)_2_D_3_ was present TGFβ and to a greater extent Th17 conditions, enhanced CTLA-4 above the level induced by 1,25(OH)_2_D_3_ alone (Fig 3a and S2 Table). In addition to CTLA-4, we analysed the effect of 1,25(OH)_2_D_3_ under cytokine treatments upon other markers known to be sensitive to 1,25(OH)_2_D_3_, including FoxP3, CD25, IL-2, IL-17, IFNγ, IL-21, and IL-10 (Fig 3b–3h and S1 and S2 Tables). 1,25(OH)_2_D_3_ did not significantly affect the overall expression of FoxP3 or IL-2 but it did alter the magnitude of the cytokine effects (P interaction <0.05). Nonetheless, in both the presence and absence of 1,25(OH)_2_D_3_ inflammatory cytokines reduced the TGFβ driven increase of FoxP3 and IL-2. For CD25, 1,25(OH)_2_D_3_ significantly increased expression across treatments but no interaction was detected. By contrast, across all cytokine backgrounds, 1,25(OH)_2_D_3_ significantly inhibited IL-17, IFNγ and IL-21 and for IL-17 an interaction between 1,25(OH)_2_D_3_ and cytokine treatment was detected, as 1,25(OH)_2_D_3_ reduced the extent of IL-17 up-regulation by TGFβ and Th17 treatments. For IL-10, which we and others have previously shown to be induced by 1,25(OH)_2_D_3_, we detected a significant effect of 1,25(OH)_2_D_3_ across cytokine treatments as well as a strong interaction. Single factor analysis also indicated that TGFβ and Th17 conditions strongly suppress IL-10 in the absence of 1,25(OH)_2_D_3_, with Th17 conditions promoting strongest suppression. Under Th0, 1,25(OH)_2_D_3_ supressed IL-10. However, in the presence of 1,25(OH)_2_D_3_ both TGFβ and Th17 conditions increased IL-10. Thus, taken together, these data demonstrate that 1,25(OH)_2_D_3_ promotes a regulatory phenotype, with high CTLA-4, and CD25 and lack of inflammatory cytokines such as IL-17, IFNγ and IL-21. In the presence of TGFβ, FoxP3 was also enhanced and 1,25(OH)_2_D_3_ raised IL-10. Most importantly, our data also show that even when inflammatory cytokines are present, 1,25(OH)_2_D_3_ has the dominant effect and a regulatory T cell phenotype is maintained.

### Vitamin D receptor expression is increased by TGFβ and maintained in the presence of Th17 polarising cytokines

**Fig 4:**
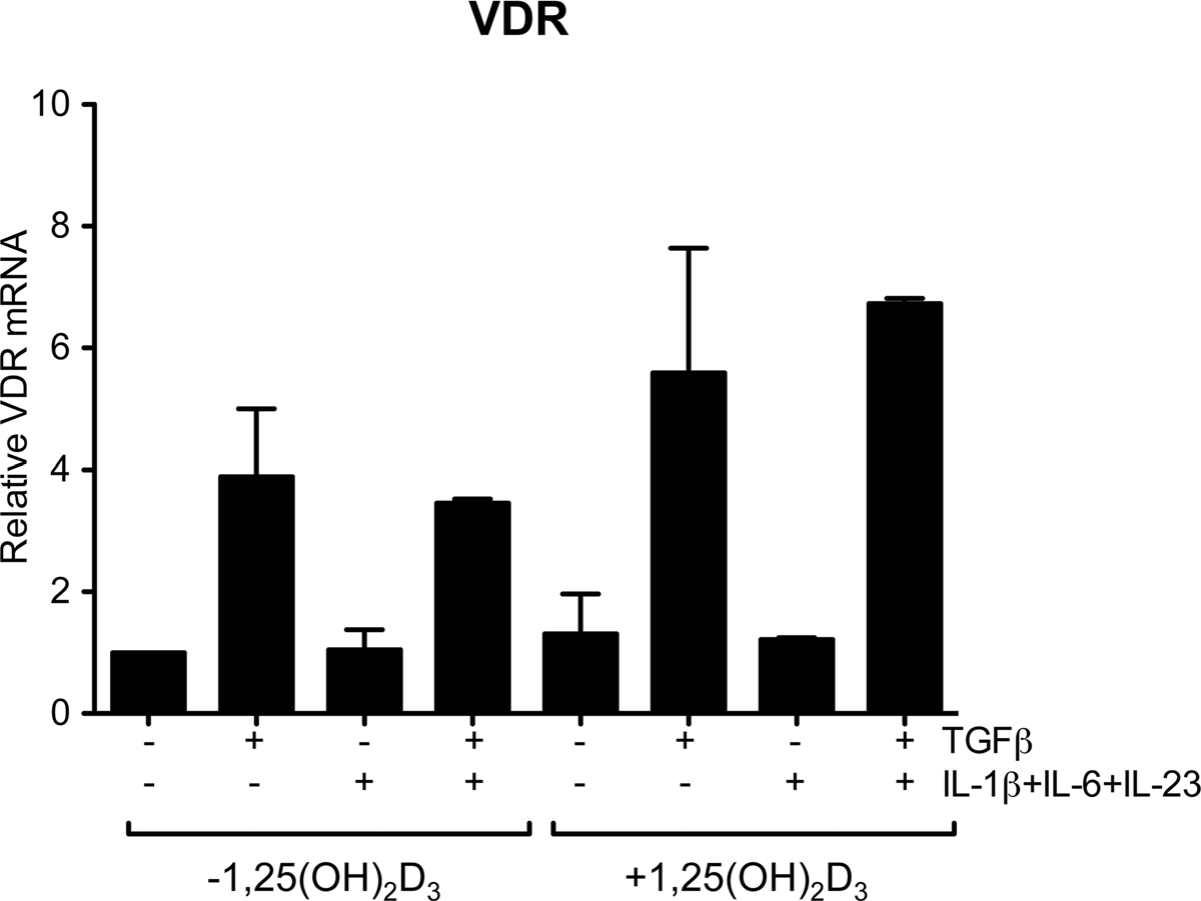
Vitamin D receptor (VDR) expression is increased by TGFβ in the presence of Th17 polarising cytokines. CD4+CD25- T cells were stimulated with antiCD3CD28 antibodies for 18 hours in the presence of recombinant cytokines and 1,25(OH)_2_D_3_ as shown and VDR mRNA measured by qPCR. Expression was normalized to 18S RNA and is plotted relative to expression in the absence of recombinant cytokines or 1,25(OH)_2_D_3_. Data are from four donors. Bars show median values and error bars indicate semi interquartile range.

Since 1,25(OH)_2_D_3_ exerts its effects through the steroidal nuclear vitamin D receptor (VDR), we investigated the impact of the above treatments on VDR expression. As shown in Fig 4, TGFβ increased VDR mRNA relative to control. This effect was maintained under Th17 conditions but inflammatory cytokines, IL-1β, IL-6 and IL-23, did not enhance VDR without TGFβ. Together these data support the hypothesis that increased VDR expression, seen under Th17 conditions, is the result of the influence of TGFβ. Moreover this suggests that the enhanced effect of 1,25(OH)_2_D_3_ on CTLA-4 expression under Th17 conditions, compared with Th0 conditions, may involve increased VDR expression.

### 1,25(OH)_2_D_3_ promotes the suppressive function of CTLA-4 under Th17 conditions

**Fig 5:**
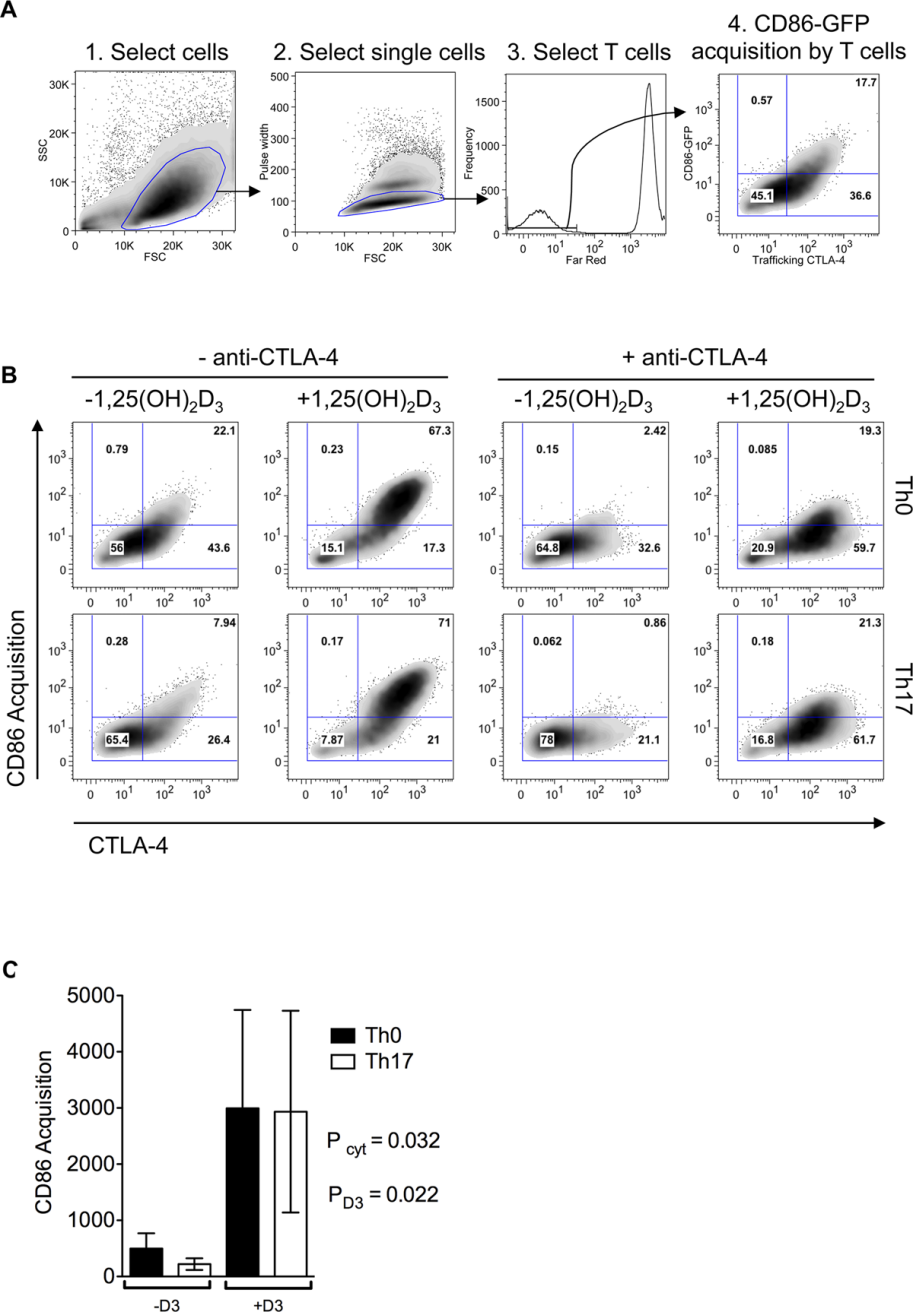
Transendocytic function of CTLA-4 is promoted by 1,25(OH)_2_D_3_ and maintained under inflammatory conditions. CTLA-4 transendocytic function was tested as described in the methods. **A)** Gating strategy to ensure exclusion of CD86-GFP donor cells from the measurement of T cell GFP acquisition. **B)** Representative FACS plots of CD86-GFP acquisition versus trafficking CTLA-4. **C)** Total CTLA-4 mediated CD86 acquisition by T cells summarized for n = 5 donors. Bars indicate mean values and error bars show the standard deviation. P values for the separate effects of Th17 cytokines (P_cyt_) and 1,25(OH)_2_D_3_ (P_D3_) are shown as determined by the 2 factor without interaction model since no interaction was detected (P = 0.146).

Transendoytosis is the major mechanism via which CTLA-4 mediates T cell suppression. Thus, having observed that Th17 cytokines suppress CTLA-4, whilst 1,25(OH)_2_D_3_ strongly enhances it, we wished to determine how these treatments affected CTLA-4 trafficking and transendocytosis. For this, we used an *in-vitro* system in which Chinese Hamster Ovary (CHO) cells, stably expressing GFP-tagged CD86, were dye-labelled and co-cultured with CTLA-4 expressing T cell blasts. Anti-CD3 was included to promote T cell activation and stimulate transendocytosis (Fig 5a). Bivariate flow cytometry analysis of CTLA-4 versus CD86 capture clearly revealed that capture of CD86 by T cells was related to the level of CTLA-4 trafficking (Fig 5b). Accordingly, the percentage of T cells that acquired CD86 was reduced under Th17 conditions (P = 0.016, Wilcoxon test n = 7). In contrast, ligand capture was substantially enhanced in the presence of 1,25(OH)_2_D_3_ even under Th17 conditions (Fig 5c). Overall, these data reveal that whilst pro-inflammatory Th17 conditions reduce CTLA-4 expression and therefore transendocytic function, 1,25(OH)_2_D_3_ overrides this effect on both expression level and on transendocytosis, driving a T cell phenotype that has high capacity to remove CTLA-4 co-stimulatory ligands from target cells.

### 1,25(OH)_2_D_3_ enhances CTLA-4-mediated suppression in dendritic cell-driven stimulations

**Fig 6:**
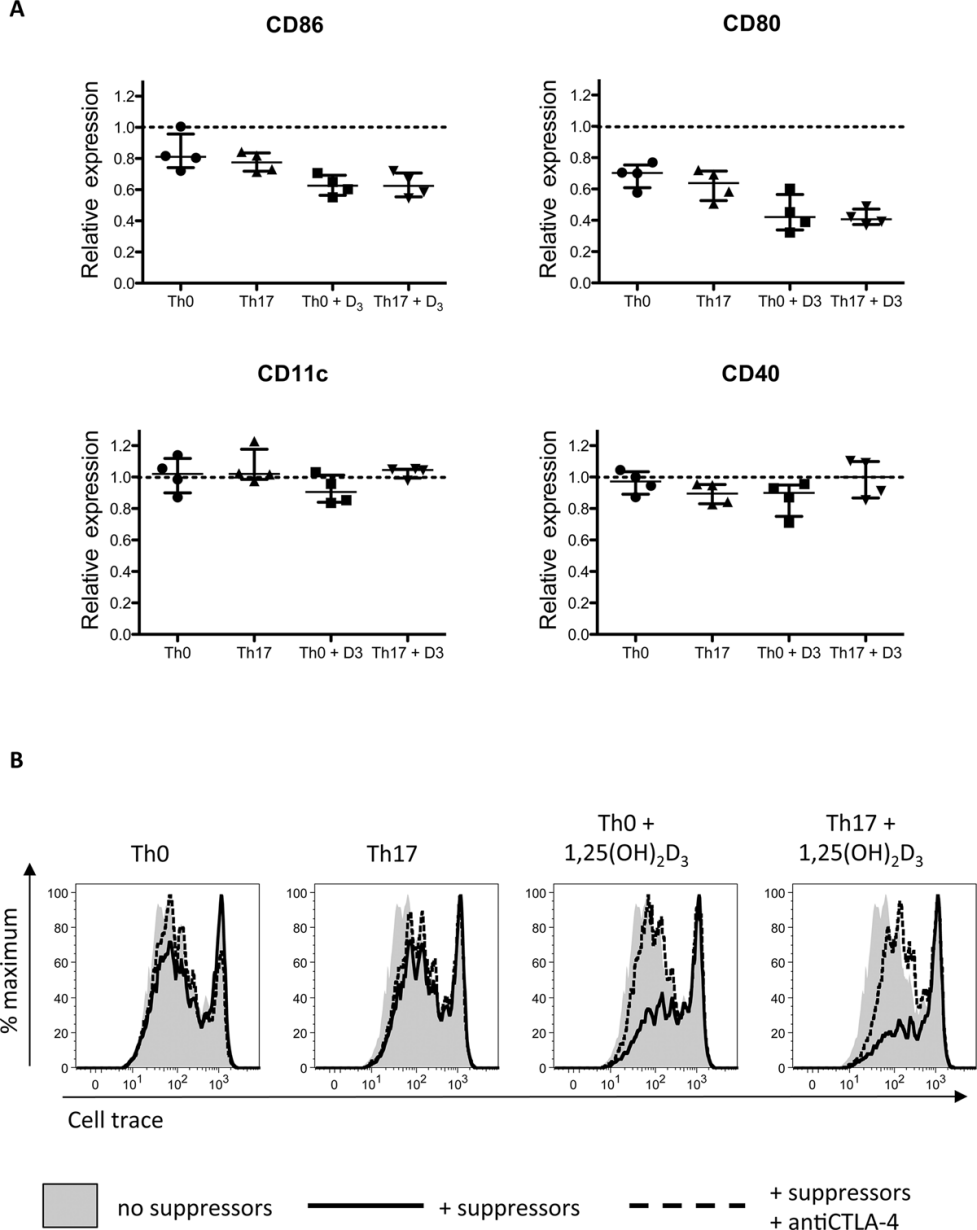
1,25(OH)_2_D_3_ promotes CTLA-4-mediated B7 depletion from dendritic cells and suppression of T cell proliferation. CTLA-4 expressing ‘suppressor cells’ were prepared by stimulating CD4+CD25- T cells under Th0 or Th17 conditions in the presence or absence of 1,25(OH)_2_D_3_. **A)** Suppressor cells were cultured with autologous DCs and antiCD3 for 24 hours with or without CTLA-4 blocking antibody. Expression of CD80, CD86, CD11c and CD40 by DCs was measured by flow cytometry. Dot plots show the ratio of marker expression in control versus anti-CTLA-4-treated cultures for four donors. **B)** Suppressor cells were CFDA-SE labeled and added to autologous DC plus antiCD3 stimulations of allogeneic cell trace violet labeled CD4+CD25- T cells (responders) with or without anti-CTLA-4. Parallel stimulations were also prepared in which CFDA-SE-labeled CD4+CD25- were added in place of suppressor T cells as a control for cell number. At five days, proliferation of responder T cells was assessed by flow cytometry. Data are from one donor but representative of four. Shaded histograms show proliferation in the absence of suppressors. Dotted and solid lines indicate proliferation in the presence versus the absence of anti-CTLA-4 respectively.

Although CTLA-4-dependent ligand capture by T cells is an important indicator of suppressive capability, it does not necessarily reflect the level of ligand downregulation on the APC. Rather, effective ligand downregulation and therefore suppression ultimately integrates a number of parameters besides CTLA-4 expression, for example ligand synthesis rates, which could be influenced by cytokines such as IFNγ and IL-10 [35–37], both of which were modified by 1,25(OH)_2_D_3_ (Fig 3c). With this in mind, we sought to test the significance of altered CTLA-4 levels, as controlled by inflammatory cytokines and 1,25(OH)_2_D_3_, upon ligand expression by co-cultured dendritic cells. T cells were stimulated under Th0 and Th17 conditions in the presence or absence of 1,25(OH)_2_D_3_, and then cultured overnight with DCs. DC expression of CD80, CD86, and the control markers CD11c and CD40 was then assessed by flow cytometry. As shown in Fig 6a, all activated T cells caused some downregulation of CD86 and CD80 but not control proteins. However, the magnitude of CTLA-4-dependent depletion was greatest for T cells cultured in the presence of 1,25(OH)_2_D_3_. Notably, depletion of CD80 was greater than that of CD86 in line with the higher affinity of CTLA-4 for CD80 [38]. Finally, we tested the ability of T cells cultured under Th17 and 1,25(OH)_2_D_3_ conditions to act as suppressor cells and affect the proliferation of responder T cells stimulated by DCs. Here we observed that Th0 and Th17 T cells had little effect upon the proliferation of responder T cells, with Th17 conditioned cells showing the least CTLA-4 mediated control. By marked contrast, T cells stimulated in the presence of 1,25(OH)_2_D_3_ under Th17 conditions as well as under Th0 conditions caused robust CTLA-4-mediated suppression (Fig 6b). Taken together, these data support the view that CTLA-4 on activated T cells plays a role in regulating co-stimulation and that 1,25(OH)_2_D_3_ enhances this pathway. Furthermore, they confirm that the effect of 1,25(OH)_2_D_3_ upon this pathway is maintained, even under inflammatory conditions.

## Discussion

A prominent feature of many autoimmune diseases is the inappropriate activation of T cells. T cell proliferation is initiated following TCR stimulation and supported by engagement of co-stimulatory CD28 with its ligands CD80 and CD86. Signals through CD28 are important for initiating and maintaining T cell proliferation, since they can overcome the induction of anergy [39]. How regulation through the CD28 axis is altered in settings of autoimmunity is therefore an important area of study. Expression of CTLA-4 is a major mechanism by which Treg cells elicit tolerance and the levels of CTLA-4 are higher in Foxp3 expressing T cells than in conventional activated T cells [40–42]. Nonetheless, expression of CTLA-4 by activated effector T cells may also be important for some aspects of immune homeostasis. Indeed, the role of CTLA-4 in non-Tregs has recently been highlighted by two studies, which reveal a suppressive capability for activated T cells *in-vivo* [43, 44]. Altered expression of CTLA-4 by effector T cells could therefore affect the duration of a T cell response. In line with this, there are differences in disease kinetics between mice that lack CTLA-4 completely and those where CTLA-4 is deficient only in Treg [42, 45, 46]. In view of these studies it is of interest to understand how CTLA-4 is regulated.

It is widely recognised that Th17 cells are key players in the development of many inflammatory disorders [18–20]. Thus we were interested in how Th17 polarising conditions would affect CTLA-4 expression and function. We observed that a pro-Th17 cytokine cocktail of TGFβ, IL-1β, IL-6 and IL-23 reduced CTLA-4 expression in CD4+CD25- T cells after activation and impaired their ability to remove co-stimulatory ligands by transendocytosis. Notably, this suppression of CTLA-4 was not restricted to Th17 cells but was evident across T cell classes including Foxp3+ in-vitro induced Tregs as well as T cells expressing IFNγ, IL-21, TNFα and IL-2. Thus suppression of CTLA-4 by pro-Th17 cytokines appears a general phenomenon that is not a consequence of the Th17 differentiation program. Physiologically, inflammation-driven down-regulation of CTLA-4 might be important to prolong T cell proliferation. Whilst this effect might be required for the efficient clearance of certain pathogens, down regulation of CTLA-4 at autoinflammatory sites could contribute to the persistence of the disease. In support of this hypothesis and our finding that Th17 conditions suppress CTLA-4 in FoxP3+ as well as FoxP3- T cells, a number of in-vivo studies have reported reduced CTLA-4 expression on Tregs and non-Tregs from patients with inflammatory disease [47–49]. Most importantly, their lack of CTLA-4 accounted for their loss of suppressive function [47].

Epidemiological studies suggest an inverse relationship between vitamin D status and the incidence of autoimmune diseases (reviewed in [25, 26, 29]). The significance of vitamin D in immune regulation is supported by the fact that immune cells, especially antigen presenting cells, express the enzyme 1α-hydroxylase that is necessary for vitamin D activation [50–53]. Indeed we have shown that activated T cells can induce 1α-hydroxylase activity in DCs at a level sufficient to influence the T cell response [51]. It has long been considered that the principal mechanism by which 1,25(OH)_2_D_3_ suppresses T cell responses is by down-regulating MHC and co-stimulatory molecules on APCs [54–56]. However, our previous finding that 1,25(OH)_2_D_3_ increases CTLA-4 through direct effects upon the T cell, suggested that the suppressive effect of 1,25(OH)_2_D_3_ upon T cell activation might be further enhanced through CTLA-4-mediated removal of CD80/86 by transendocytosis. Through this study we have confirmed that by increasing CTLA-4, 1,25(OH)_2_D_3_ can increase transendocytic removal of CD80/86 from APCs and substantially reduce their ability to stimulate T cells. Thus, 1,25(OH)_2_D_3_ appears to target both the APC and T cell sides of the CD80/CD86-CD28/CTLA-4 axis to achieve maximal suppression of T cell responses. Importantly we have also shown that this ability of 1,25(OH)_2_D_3_ to increase CTLA-4 expression and transendocytic function is retained, if not enhanced, under Th17 polarising conditions. Taken together these data suggest that low levels of vitamin D might predispose to exaggerated inflammatory reactions along with impaired CTLA-4 expression and that provision of 1,25(OH)_2_D_3_ would reverse this effect even at sites of active Th17-driven inflammation.

Many chronic inflammatory diseases develop as a consequence of exposure to environmental triggers in a genetically susceptible individual. Interestingly, a number of polymorphisms associated with chronic inflammatory diseases lie within the co-stimulation gene locus at chromosome 2q33 that encodes CD28, CTLA-4 and ICOS [57–61] and ChIP-seq studies have revealed the presence of VDR binding sites near to these polymorphisms [62]. Thus regulation of CTLA-4 expression could be a point at which genetic and environmental factors interact to influence an immune response and the establishment of disease.

Overall, our study has provided new insights into the regulation of CTLA-4, a critical suppressive protein whose loss of function is associated with autoimmunity. In particular we have shown that inflammatory Th17 conditions, suppress the expression and transendocytic function of CTLA-4, but demonstrate that their effect can be opposed by 1,25(OH)_2_D_3_. In addition, we confirmed that the ability of 1,25(OH)_2_D_3_ to suppress inflammatory cytokines and increase IL-10 was retained even under inflammatory conditions. Our findings therefore highlight a potential role for vitamin D as an environmental factor in the control of autoimmunity and support the exploration of its use as a therapeutic agent for established disease as well as in prophylaxis.

## Supporting Information

S1 Fig Effect of Th17 polarising cytokines upon CTLA-4 expression. Cell trace-labelled CD4+CD25- T cells were stimulated for four days with antiCD3CD28 beads in the presence of recombinant cytokines TGFβ, IL-1β, IL-6 and IL-23 as indicated and expression of total CTLA-4 assessed by flow cytometry. Expression is given relative to the level in non-treated T cells.
(TIF)

S1 Table Summary of within subject effects and contrasts as determined by repeated measure two factor within subject analysis for regulatory and inflammatory associated T cell markers. (PDF)

S2 Table Summary of within subject effects and contrasts as determined by repeated measure single factor within subject analysis for regulatory and inflammatory associated T cell markers. (PDF)
