# Supplementary figures and images for: Vitamin D Antagonises the Suppressive Effect of Inflammatory Cytokines on CTLA-4 Expression and Regulatory Function
Source: PLoS One. 2015 Jul 2;10(7):e0131539. doi: 10.1371/journal.pone.0131539 (PMC4489761; doi:10.1371/journal.pone.0131539)

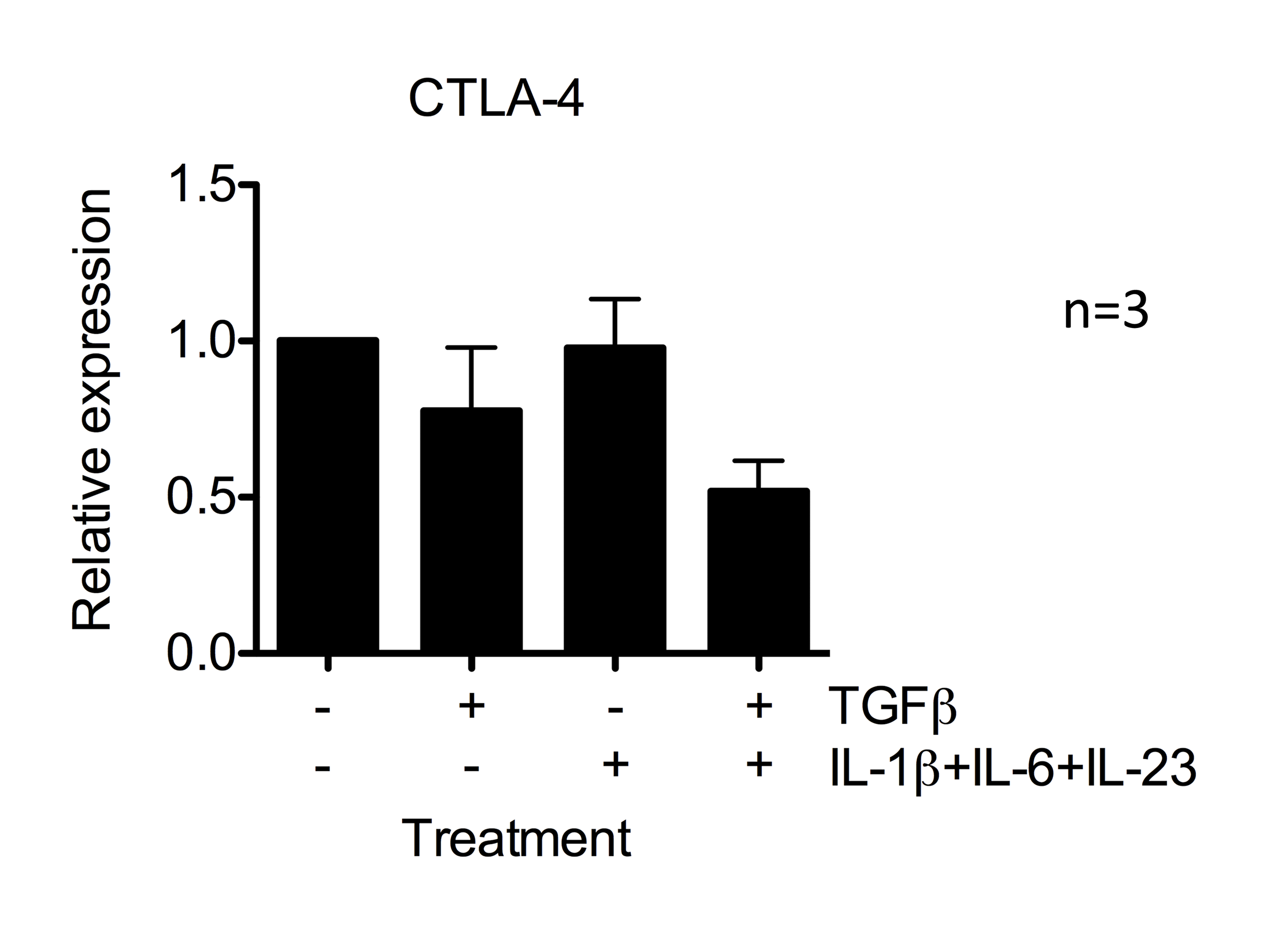

Supplement: S1 Fig — Cell trace-labelled CD4+CD25- T cells were stimulated for four days with antiCD3CD28 beads in the presence of recombinant cytokines TGFβ, IL-1β, IL-6 and IL-23 as indicated and expression of total CTLA-4 assessed by flow cytometry. Expression is given relative to the level in non-treated T cells. (TIF) [file pone.0131539.s001.tif]
