# Supplementary material for: Vitamin D Antagonises the Suppressive Effect of Inflammatory Cytokines on CTLA-4 Expression and Regulatory Function
Source: PLoS One. 2015 Jul 2;10(7):e0131539. doi: 10.1371/journal.pone.0131539 (PMC4489761; doi:10.1371/journal.pone.0131539)
Supplement: S1 Table — (PDF) [file pone.0131539.s002.pdf]

S1 Table: Summary of within subject effects and contrasts as determined by repeated measure 2 factor within subject analysis for regulatory and inflammatory associated T cell markers.

	Factor								
	Cytokine				$1,25(OH)_2D_3$	Cytokine * $1,25(OH)_2D_3$ Interaction			
	Overall Effect (P_{Cyt})	Contrasts			Overall effect (P_{D3})	Overall	Contrasts		
Marker		Th0 vs TGFβ	Th0 vs Th17	TGFβ vs Th17			Th0 vs TGFβ	Th0 vs Th17	TGFβ vs Th17
CTLA-4	0.008	0.042	0.009	<0.001	<0.001	<0.001	0.004	<0.001	<0.009
FoxP3	<0.001	<0.001	0.023	<0.001	0.085	0.009	0.015	0.962	0.028
CD25	0.228	0.054	0.747	0.126	0.022	0.296	0.523	0.289	0.296
IL-2	0.048	0.009	0.585	0.026	0.055	0.007	0.008	0.012	0.947
IL-17	<0.001	0.001	0.001	0.001	<0.001	<0.001	0.009	0.001	0.009
IFNγ	<0.001	<0.001	0.857	<0.001	<0.001	0.600	0.585	0.307	0.688
IL-21	0.352	0.285	0.875	0.250	<0.001	0.642	0.430	0.329	0.993
IL-10	0.115	0.714	0.08	0.099	0.001	<0.001	<0.001	<0.001	0.046
