# Supplementary material for: Vitamin D Antagonises the Suppressive Effect of Inflammatory Cytokines on CTLA-4 Expression and Regulatory Function
Source: PLoS One. 2015 Jul 2;10(7):e0131539. doi: 10.1371/journal.pone.0131539 (PMC4489761; doi:10.1371/journal.pone.0131539)
Supplement: S2 Table — (PDF) [file pone.0131539.s003.pdf]

S2 Table: Summary of within subject effects and contrasts as determined by repeated measure single factor within subject analysis for regulatory and inflammatory associated T cell markers.

	Background Condition							
	$-1,25(OH)_2D_3$				$+1,25(OH)_2D_3$			
Marker	Overall effect (P_{Cyt-D3})	Th0 vs TGFβ	Th0 vs Th17	TGFβ vs Th17	Overall effect (P_{Cyt+D3})	Th0 vs TGFβ	Th0 vs Th17	TGFβ vs Th17
CTLA-4	<0.001	0.085	0.002	0.005	<0.001	0.013	0.001	0.018
FoxP3	<0.001	<0.001	0.007	<0.001	<0.001	0.001	0.088	0.033
IL-2	0.040	0.057	0.531	0.007	0.017	0.002	0.231	0.036
IL-17	<0.001	<0.001	<0.001	<0.001	<0.001	0.016	0.001	0.013
IL-10	<0.001	<0.001	<0.001	0.001	0.003	0.003	0.011	0.649
